# Laser Powder Bed Fusion Applied to a New Biodegradable Mg-Zn-Zr-Ca Alloy

**DOI:** 10.3390/ma15072561

**Published:** 2022-03-31

**Authors:** Radu Emil Hendea, Doina Raducanu, Anna Nocivin, Steliana Ivanescu, Doina Stanciu, Corneliu Trisca-Rusu, Radu Septimiu Campian, Silviu Iulian Drob, Vasile Danut Cojocaru, Bogdan Mihai Gălbinașu

**Affiliations:** 1 Department of Oral Rehabilitation, Faculty of Dental Medicine, Iuliu Hatieganu University of Medicine and Pharmacy, 400349 Cluj-Napoca, Romania; raduhendea@yahoo.com (R.E.H.); rcampian@email.com (R.S.C.); 2 Department of Metallic Materials Processing and Ecometallurgy, University POLITEHNICA of Bucharest, 060042 Bucharest, Romania; doina.raducanu@upb.ro; 3 Faculty of Mechanical, Industrial and Maritime Engineering, OVIDIUS University of Constanta, 900527 Constanța, Romania; anocivin@univ-ovidius.ro; 4 ZIRCON DENT SRL, 400690 Cluj-Napoca, Romania; sivanescu@yahoo.com (S.I.); doinastanciu09@yahoo.com (D.S.); 5 National Institute for Research and Development in Micro-technologies, 077190 Bucharest, Romania; corneliu.trisca@nano-link.net; 6 Romanian Academy, Institute of Physical Chemistry “Ilie Murgulescu”, Spl. Independentei 202, 060021 Bucharest, Romania; sidrob.icf@gmail.com; 7 Dental Medicine Faculty, University of Medicine and Pharmacy “Carol Davila” Bucharest, 020021 Bucharest, Romania; bogdan.galbinasu@umfcd.ro

**Keywords:** magnesium alloy, laser powder bed fusion, processing parameters, structural and mechanical properties

## Abstract

The aim of the present paper is to apply the laser powder bed fusion process to a new biodegradable Mg-Zn-Zr-Ca alloy powder prepared via a mechanical alloying method from powder pure components. This additive manufacturing method is expected to allow for the obtaining of high biomechanical and biochemical performance. Various processing parameters for laser powder bed fusion are tested, with a special focus on laser energy density—E [J/mm^3^]—which is calculated for all experiment variants, and which represents an important processing parameter, dependent upon all the rest. The goal of all the trials is to find the most efficient schema for the production of small biodegradable parts for the medical domain, meaning the selection of optimal processing parameters. An important observation is that the most robust and homogeneous samples without cracks are obtained for lower values of the E, around 100 J/mm^3^. Thus, the most performant samples are analyzed by scanning electron microscopy, X-ray diffraction and by compression mechanical test.

## 1. Introduction

In biomedical applications, the most commonly used metallic materials are still stainless steel, cobalt chromium alloys and titanium and its alloys [1]. They have a disadvantage with regard to the contamination potential of tissue due to metal corrosion and/or abrasion, which may decrease medical device biocompatibility, leading to the loss of tissue [2]. Implants made of these metallic materials have an elastic modulus, which does not match that of bone tissue, leading to a stress effect that reduces the stimulation of new bone growth and repair, which decreases implant stability. Once the bone fracture has totally healed, these implants may be removed using an invasive secondary surgery [3], especially recommended for young people and growing children. 

Biodegradable metallic materials for stents, orthopaedic and maxillofacial temporary implants have attracted extensive attention in recent years. Their good combination of biodegradability and biomechanical properties can make a second surgery, which is painful for patients and costly for countries’ health systems, not necessary. Considered as revolutionary metallic biomaterials for implantology, magnesium and its alloys have received many research and clinical investigations in the last years due to their important advantages. Firstly, the mechanical properties are close to natural bone, thus avoiding the effect of stress shielding caused by the mismatch of the elastic modulus. Magnesium products have a Young’s modulus of 41–45 GPa, which, compared to that of human bone (3–20 GPa), is closer than that of well-known permanent implant materials such as stainless steel (190–205 GPa), titanium and its commercial alloys (110–117 GPa) and cobalt chromium alloys (230 GPa) [4]. Secondly, magnesium has a very high biocompatibility, being an essential element, the fourth-most abundant metal element in the human body and very important for the human metabolism. Moreover, studies have highlighted that magnesium ions can promote bone healing and the formation of new bone [5].

Investigations of the in vivo degradation of some Mg-based implants, such as screws for mandibular fracture and cardiovascular stents made of Mg–Nd–Zn–Zr alloy, have proved their potential for medical implantation. Nevertheless, these Mg-based implants produced through conventional manufacturing processes (melting, thermo-mechanical processing and machining) showed limitations in regard to the obtaining of complex geometries and desirable biomechanical properties, non-negligible disadvantages in particular for patient-customized implants and tissue engineering scaffolds [5].

An ideal bone substitute might have, besides biocompatibility, a fully interconnected porous structure, which allows bone ingrowth and has bone-mimetic mechanical properties, thus able to provide sufficient support and to avoid stress shielding. For this reason, highly porous metal implants are needed to achieve better biocompatibility properties than that of traditional solid metals with a compact structure. Having appropriately designed characteristics such as porosity, a surface coefficient and an elastic modulus (Young’s modulus), the porous structures can mimic the properties of cancellous bone [6].

In the last years, additive manufacturing (AM) has gained a great deal of attraction for different industries due to its superiority over the conventional manufacturing processes, as it allows accurate control for achieving complex geometries and/or architectures. Thus, AM techniques are promising methods by which to process biomaterials for manufacturing implants and stents used in the field of orthopaedics, craniofacial and cardiovascular surgery [5,7]. Research studies have shown that AM is a suitable technique for obtaining porous implants and bioactive scaffolds for tissue regeneration. These structures can be entirely biodegradable, depending on the scaffold material properties [7].

AM for metallic parts consists of powder metallurgy methods based on the bed fusion (PBF) of different metallic powders or powder alloys, using electron-beam (EB-PBF) or more frequently laser (L-PBF), otherwise known as *selective laser melting* (SLM). For Mg and its alloys, the EB-PBF system is not suitable due to magnesium evaporation, which affects the propagation of the electron beam in vacuum. However, it is adequate for the L-PBF technique [8]. The powder alloy is obtained by mechanical alloying of the component elements.

The L-PBF process uses one or several micro-scaled high-density laser beams that selectively scan and fuse a powder bed, layer-by-layer. The successive solidified layers stack upon each other and build a near net-shaped three-dimensional part, enabling the rapid fabrication of metallic products, with no involvement of molds or fixtures. L-PBF allows a high flexibility in geometry design and in tailoring the microstructure and performances of the manufactured parts, obtained in a shorter time than when using well-known conventional methods. Furthermore, L-PBF-fabricated components usually have a satisfactory forming accuracy, obtaining near-final shape products. In this way, AM products require only a little or no post-machining [9,10].

The first step of the L-PBF process is pre-processing, which consists of the designing of the CAD model representing the 3D geometry and its conversion to a special file format named STL (stereolithography). Using special algorithms, the 3D model is sliced into very thin layers that are usually from 20–40 μm. Next, the L-PBF machine needs the design and setting of the process parameters, as well as the layers’ distances, laser power, scan speed and hatch distance. By correlating these parameters of scanning strategy, the laser energy density can be calculated. All the above data must be appropriate for the processed material. The second step is the AM process, where the software of the L-PBF machine obtains the slicing information and scans the powder bed, layer-by-layer, following the designed geometry, until the part is completely built [10].

The final characteristics of solid, porous or scaffold products obtained by L-PBF depend on the applied AM parameters and flexible manufacturing design. The L-PBF technique enables the improvement and/or tailoring of the mechanical properties of metallic products, impossible to be performed upon wrought and as-cast products. The good mechanical properties associated with designed porosity and, most importantly, controlled corrosion characteristics, support the great potential of L-PBF application for ultralight structures, including for biodegradable metallic implants [11]. All the L-PBF advantages, proved by certain research results, recommend it as a new method for the rapid manufacturing of high-quality products, including those made of magnesium and magnesium alloys [12]. Until now, research works have focused only upon a limited number of L-PBF-processed Mg-based biomaterials. Those relevant are: pure Mg [12]; binary Mg alloys such as Mg-Al [9,13], Mg-Ca [14] and Mg-Zn [15]; ternary alloy systems such as AZ (Mg-Al-Zn) [16] and ZK (Mg-Zn-Zr) [17] series; Mg alloys with rare earth (RE) elements such as WE43 (Mg-Zr-RE-Y) [2] and Mg-Zn-Dy [18]; Mg-Zn-Zr-Nd alloys [5]; Mg–Y–Sm–Zn–Zr [19]; and Mg-11.00Gd-1.77Zn-0.43Zr [20]. Several pure Mg parts produced by the L-PBF technique have an elastic modulus in the range of 27–33 GPa, more closely matched with that of human bone compared with other metallic biomaterials [12].

The L-PBF-processed ZK60 powder alloy has exhibited higher corrosion resistance in Hanks’ solution than the as-cast ZK60 alloy, a decrease of hydrogen evolution rate of about 30% and about a 50% lower corrosion current density [16].

Investigation of the in vitro degradation behaviour and mechanical properties of WE43 scaffolds, obtained by the L-PBF processing of powder alloy, shows mechanical properties high enough for adequate mechanical support and a satisfactory degradation rate (20% volume loss after 4 weeks of biodegradation) [17].

The L-PBF-processed Mg-11.00Gd-1.77Zn-0.43Zr alloy has a yield strength (YS) of 325 MPa, an ultimate tensile strength (UTS) of 332 MPa and an elongation of 4.0% at room temperature. Comparing with as-cast alloys, L-PBF-ed samples have almost similar elongation (+0.4%) though higher YS (+162 MPa) and UTS (+122 MPa) [19].

Most research studies on Mg-based biodegradable alloys processed using the L-PBF technique are preliminary attempts, which refer to magnesium alloys from different systems: Mg-Al-Zn (AZ), Mg-Zr-RE-Y, Mg-Zn and Mg-Zn-Zr with or without Y, Sm, Gd, Nd, etc. Biological investigations found that Mg-Al-Zn alloy systems must be avoided, because in the human body released Al ions from implants are associated with neurotoxic disorders (Alzheimer’s). The WE alloy series, despite their good bio-corrosion resistance, may cause severe hepatotoxicity due to the RE content [21].

Among the studied compositions, the ZK (Mg-Zn-Zr) alloys series attracted more attention from researchers due to their composition with a good biocompatibility of the all-component elements. Thus, ZK alloys are more attractive than AZ and WE in terms of element biocompatibility for use as biodegradable and biosafety candidates for bone repair implants [22]. However, drawbacks still remain related to the degradation rate and mechanical integrity of the implants until bone healing. 

Therefore, for our work, the composition of the biodegradable magnesium alloy was established in the multicomponent system Mg-Zn-Ca-Zr, the alloying elements being highly biocompatible, i.e., biodegradable essential elements (Mg, Zn and Ca) or bio-inert (Zr). Previously, we experimented with some alternative compositions with different Zn contents, selecting a new promising composition for biodegradable implants, Mg-10Zn-0.8Ca-0.5Zr (%wt.) [8], for L-PBF processing and obtaining good mechanical characteristics and adequate degradation in simulated body fluids. The Mg-Zn-Ca-Zr alloy for L-PBF processing was prepared from the component metal powders by mechanical alloying. 

The goal of this work was to study the L-PBF processability of this new Mg alloy and the structural and mechanical characteristics of the AM samples. Our research strategy provided variation of the L-PBF process parameters (laser power, scanning speed, layer thickness and specific laser energy density) and found a good combination of them which avoided the main drawbacks related to the L-PBF process, such as oxidation, smog, balling, crack formation, delamination or a loss of alloying elements that affects the quality of the AM product.

## 2. Materials and Methods

### 2.1. Mechanical Alloying for Preparing the Mg-Zn-Ca-Zr Powder Alloy

The feedstock for the L-PBF process is a powder alloy prepared by mechanical alloying (MA) of alloy component powders. The new composition of biodegradable alloy Mg-10Zn-0.8Ca-0.5Zr (%wt.) was established in the previous paper of the present authors [8], in which the MA parameters were defined. Thus, after a series of MA tests for three different alloy compositions of the system Mg-Zn-Ca-Zr, the different tested MA processing schema with variable milling parameters were described in detail. The conclusion of this previous work was that the most suitable/promising variant for further application of the L-PBF in the case of a powder alloy with the above chemical composition has the following MA parameters: 300 rpm for the milling speed, 28 h for the milling time, 10:1 for the powder to oxide ball weight ratio and sieving between 30 and 60 µm at the end. Mechanical alloying was carried out using a high-energy PM 100 Retsch planetary mill (Retsch, Haan, Germany) (500 mL capacity/10 mm diameter of zirconium oxide balls) under an argon atmosphere—1.5 bar overpressure.

**Figure 1:**
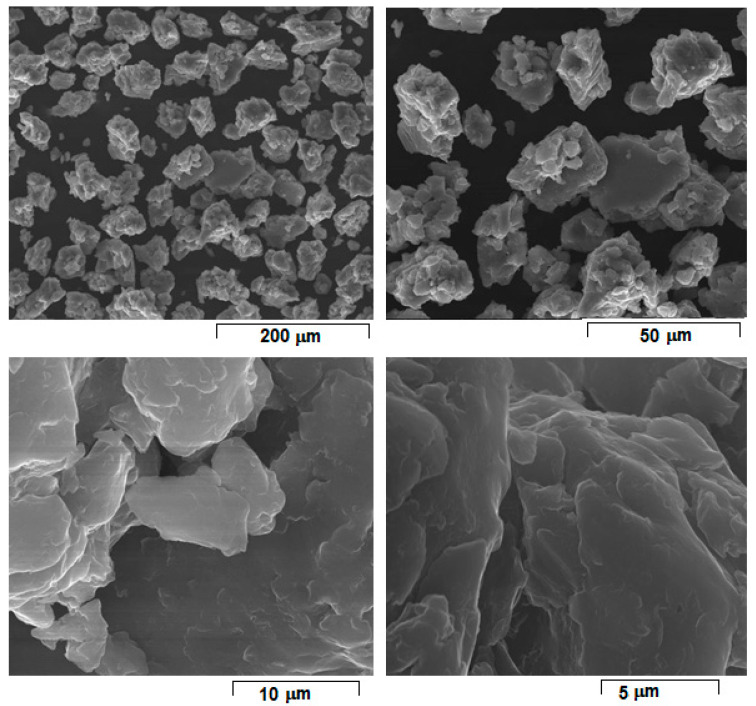
The SEM-SE images of the Mg-10Zn-0.8Ca-0.5Zr powder alloy obtained by mechanical alloying with 300 rpm/28 h/10:1 applied parameters and final sieving between 30–60 µm; different magnifications.

Figure 1 shows the morphology of the initial powder alloy prepared with the above selected parameters for L-PBF processing. The powder consists of majority α-Mg solid solution phase, a fact additionally supported by XRD analysis [8].

As a general remark, the choice of optimal powder obtaining method for L-PBF (as MA is used for the present case) involves considering a series of complex aspects that are to the advantage or disadvantage of each possible variant. At the moment, none of these variants (i.e., gas or water atomization, mechanical alloying or other technologies) are recommended more or less due to pro and contra arguments provided [23,24]. What is already established is that the metallic powder necessary for the L-PBF should be as fine as possible, a uniform size, high density, without pores or inclusions and, if possible, of spherical shape, all to assure best concordance between designed geometry and as-built geometry [23,24]. Apparently, gas atomization and mechanical alloying could both be suitable for this purpose, but in terms of resource consuming (materials and energy) on the entire processing chain, mechanical alloying is reported to be quite efficient and a low-cost modality for producing metallic powders with the above characteristics [25]. In addition, it should be considered that due to repeated fracturing and cold welding of the particles caused by severe plastic deformation, mechanical alloying leads to a powder material with a nano-crystalline structure even if the powder shape is not so regular and spherical as it is with gas atomization [26,27,28].

### 2.2. L-PBF Processing of the Mg-Zn-Ca-Zr Powder Alloy

Previous L-PBF trials [8] on Mg-10Zn-0.8Ca-0.5Zr (%wt.) powder alloy, obtained by MA with the above applied parameters, were conducted upon robust samples, non-friable and without cracks. However, these incipient L-PBF trials are insufficient for a consistent conclusion regarding an assumed schema of L-PBF processing parameters that can assure quality and reproducible samples with satisfactory results. This is the reason why, in this work, the L-PBF trials were extended for more systematic variation and testing of the L-PBF processing parameters in order to establish the most suitable combination of parameters. 

**Figure 2:**
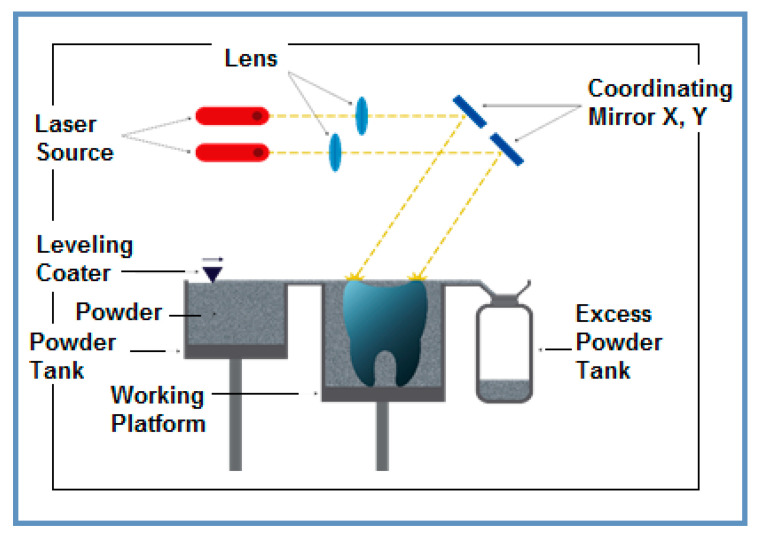
System MySint100 Dual Laser for additive manufacturing.

The type of laser used was MYSINT 100-3D Selective Laser Fusion (SISMA s.p.a., Vicenza, Italy)—a printer for metal powder, with power supply 220–240 V, 50/60 Hz; maximum power absorbed 1.9 kW; inert gas—Nitrogen, Argon (Figure 2).

The technological flow includes: computer aided design (CAD) of the product model (implant or sample of Mg alloy) using specialized software and saving of data in an STL file (stereolithography), followed by computer aided machining (CAM) consisting of: transferring data from the STL file for splitting and then transferring to the laser printer, programming the L-PBF parameters, AM of the product and detaching the AM product from the building plate.

Table 1 shows the intervals of the applied L-PBF processing parameters. From these intervals, different parameter combinations (various laser scanning strategies) for 15 distinct variants tests have been selected. 

For each of these 15 tested variants, E—the laser energy density [J/mm^3^] was calculated, which represents an important processing parameter, dependent upon all the rest and through which qualitative sample densification, microstructure and mechanical properties of the final 3D product can be assured. The used laser energy density (E) calculation formula is [29]:

(1) $$E=\frac{Laser\;power}{Scanning\;speed\;\times \;Hatch\;spacing\;\times \;Layer\;height}$$

### 2.3. Microstructural and Mechanical Investigations of the L-PBF-Processed Mg-Zn-Ca-Zr Powder Alloy

Scanning electron microscopy–secondary electron (SEM-SE) imaging investigations were performed using a Tescan VEGA II-XMU SEM microscope (Tescan Orsay Holding, a.s., Brno, Czech Republic). This analysis was performed to examine the characteristics of the obtained powders and SLM-ed samples: their consistency/density, homogeneity, morphology, dimension, no/yes cracks, porosity, etc. The SEM examination was made in the fracture area, inside the L-PBF samples, upon different spatial directions. A conventional metallographic examination on a polished surface was also performed following conventional procedures: the samples were embedded in XPHC phenolic conductive resin (NX-MET, Echirolles, France); a Metkon Digiprep ACCURA type machine (Metkon Instruments Inc., Bursa, Turkey) was used to abrade the samples with SiC 240 to 1200 grit papers (NX-MET, Echirolles, France) and to polish the samples with 6, 3, and 1 μm alcohol diamond suspensions (NX-MET, Echirolles, France) on X200 polishing pads (NX-MET, Echirolles, France).

The porosity analysis was performed using two methods: firstly, with an estimation based on direct measurement of the pores’ average dimensions on SEM images, highlighting the distribution of the pores; and secondly, using a classical method which implied the immersion of the sample in the xylene (in conformity with Romanian standard SR-ISO-2738:1994) and the determination of two sample masses saturated in xylene, one weighed in the air, and the other one weighed in xylene. Based on these two determinations, the apparent porosity value and the open porosity value can be calculated. 

Qualitative X-ray diffraction (XRD) analysis was conducted to identify the presence of structural components and phases for the obtained L-PBF samples. The conventional X-ray diffraction was carried out at room temperature (RT, 298 K) using a Panalytical X’Pert PRO MRD diffractometer (Malvern Panalytical Ltd., Malvern, UK), with Cu-kα radiation (λ = 0.15418 nm) in the 2θ range of 30°–90°, using a step size of 0.02° and an operating voltage and current of 40 kV and 30 mA, respectively. The recorded XRD patterns were fitted using the PeakFit v4.11 software package (version 4.11, Systat Software Inc., London, UK) to de-convolute the observed cumulative diffraction peaks and to determine, for each constitutive peak, the position, intensity, and broadening FWHM (full width at half maximum).

The final L-PBF samples were mechanically tested for compression. The test was performed on a universal INSTRON 3382 material testing machine (Instron Ltd., High Wycombe, Buckinghamshire, HP123SY, UK). The load was applied at an increasing rate up to the maximum load value at which the sample broke. The corresponding stress–strain curves were obtained and analyzed. 

## 3. Results and Discussion

**Figure 3:**
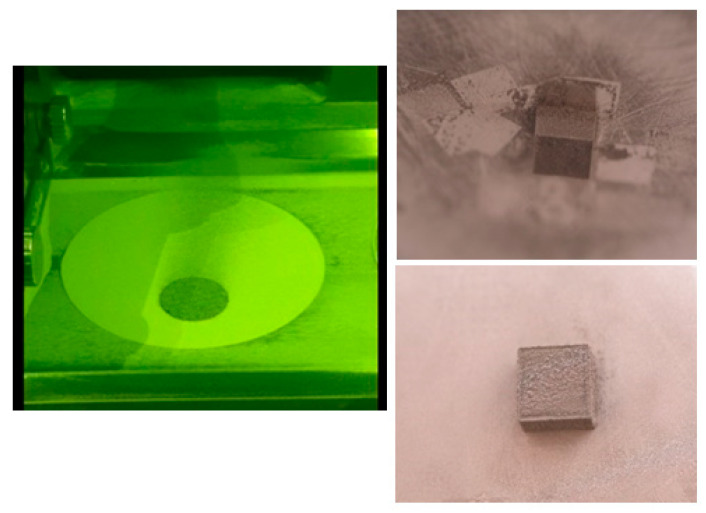
Video capture during L-PBF process (**left**). Two samples of L-PBF-processed Mg-10Zn-0.8Ca-0.5Zr powder alloy (**right**).

The obtained L-PBF samples were analyzed from a microstructural point of view. The macroscopic examination at first removed the samples with a weak compactness, that were very friable and with cracks; only robust samples with few and shallow pores and no cracks were considered for further analysis. It was not considered relevant to show the evidence with cracks. Figure 3 shows two macro examples from these suitable samples.

**Figure 4:**
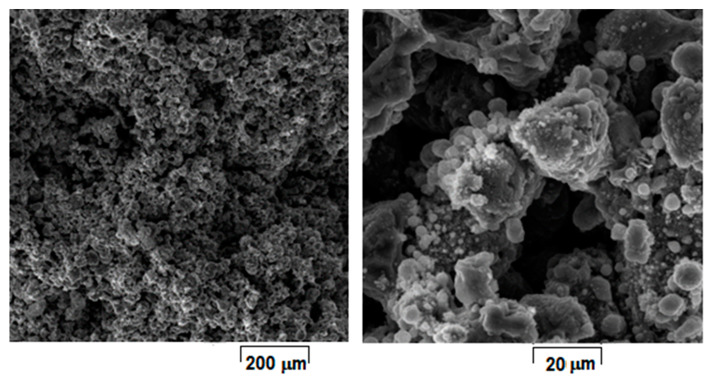
SEM-SE images of L-PBF sample obtained with laser power 180 W; scanning speed 700 mm/s; layer height 20 µm applied parameters and calculated laser energy density 429 J/mm^3^.

**Figure 5:**
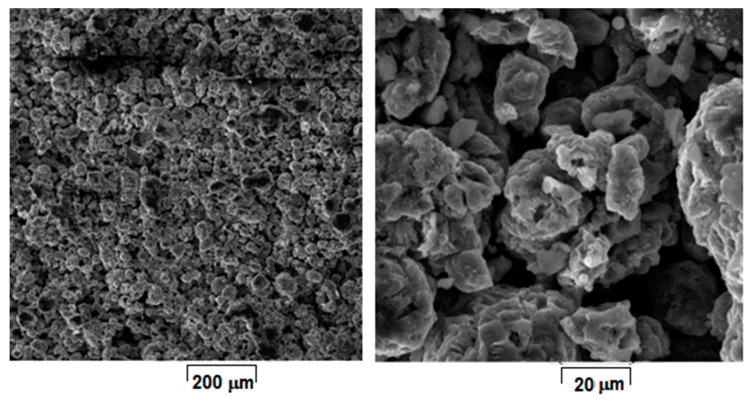
SEM-SE images of L-PBF sample obtained with Laser power 180 W; Scanning speed 1000 mm/s; Layer height 20 µm applied parameters and calculated Laser energy density 300 J/mm^3^.

**Figure 6:**
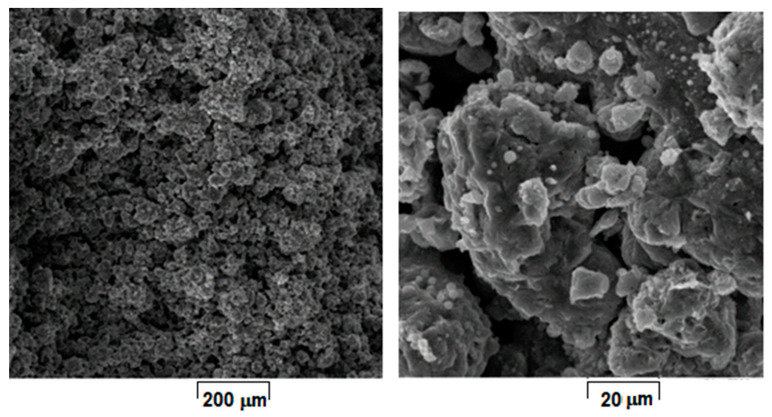
SEM-SE images of L-PBF sample obtained with laser power 200 W; scanning speed 700 mm/s; layer height 30 µm applied parameters and calculated laser energy density 238 J/mm^3^.

**Figure 7:**
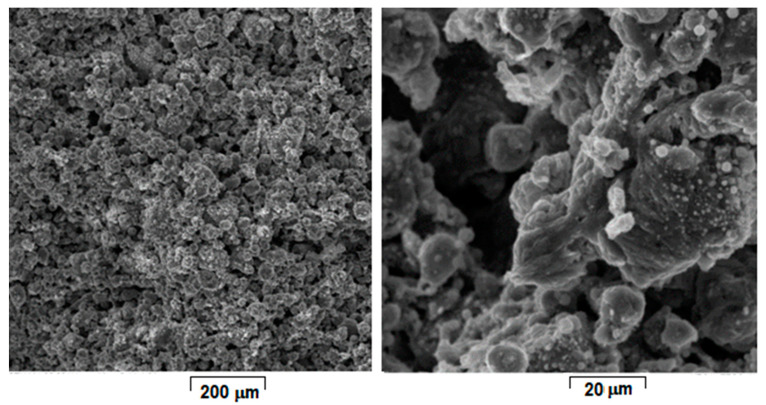
SEM-SE images of L-PBF sample obtained with laser power 175 W; scanning speed 700 mm/s; layer height 50 µm applied parameters and calculated laser energy density 167 J/mm^3^.

**Figure 8:**
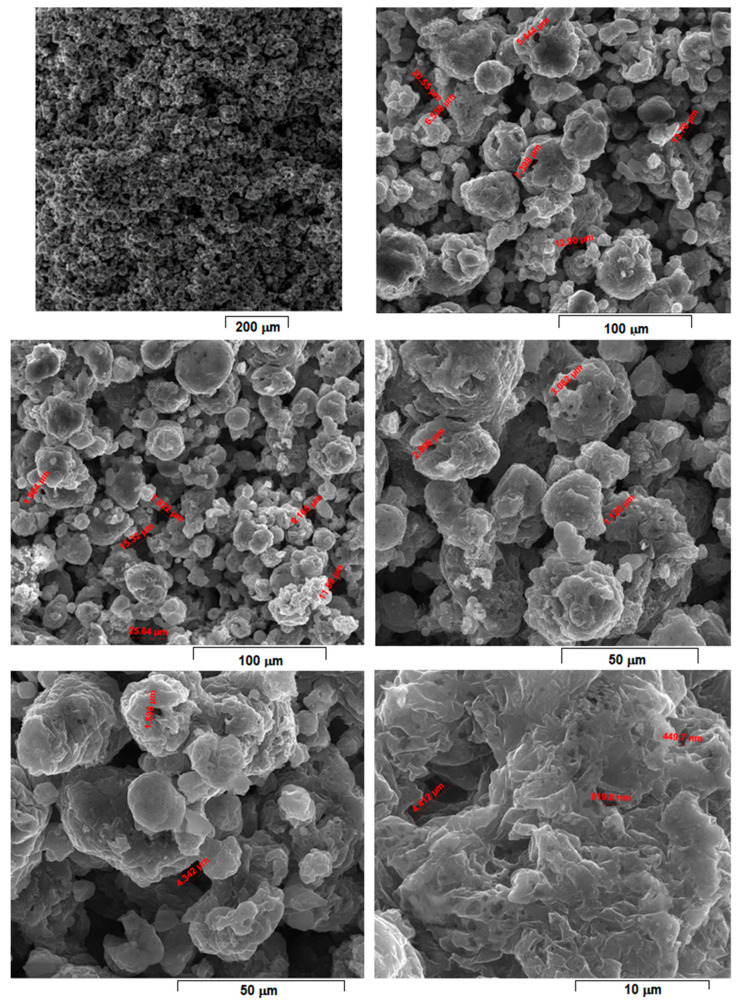
SEM-SE images of L-PBF sample obtained with laser power 150 W; scanning speed 1200 mm/s; layer height 30 µm applied parameters and calculated laser energy density 139 J/mm^3^.

**Figure 9:**
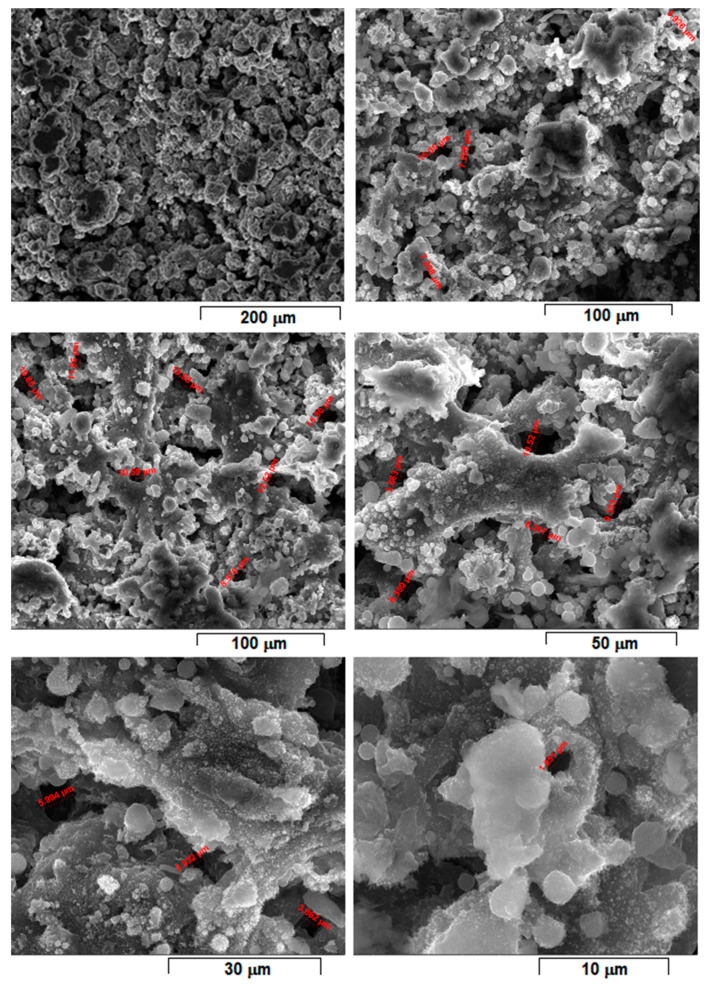
SEM-SE images of L-PBF sample obtained with laser power 45 W; scanning speed 600 mm/s; layer height 30 µm applied parameters and calculated laser energy density 100 J/mm^3^.

Considering that laser energy density (E) represents an important and influential processing parameter that cumulates all others, the SEM images from Figure 4, Figure 5, Figure 6, Figure 7, Figure 8 and Figure 9 have been selected to present the most relevant samples, as mentioned above, but in function of the E descending value order, calculated with formula (1): 429 → 300 → 238 → 167 → 139 → 100 J/mm^3^. These six samples proved to be the most resistant, with low friability of all that were obtained and tested. However, according to the initial proposed objective, the aim was to obtain a relatively porous but robust structure that can mimic the cortical bone structure. Therefore, to observe porosity consistency, the SEM microstructural analysis was performed not at the surface of the obtained samples but inside of them, in the fracture area, upon different spatial directions. 

When observing the samples in E descending order, no major differences were apparently seen: all showed a porous morphology. However, the samples with higher E (Figure 4, Figure 5, Figure 6 and Figure 7) were more friable and showed a more irregular morphology of pores compared to those with lower E (Figure 8 and Figure 9). They were less consolidated, a deficiency that diminishes as the value of E decreases. Most importantly, none of the selected samples showed the balling effect which is known to diminish the mechanical performance of the final L-PBF-processed samples [29]. Consequently, the most acceptable present results were of the sample with E = 100 J/mm^3^. Therefore, for this particular sample, an SEM-EDS analysis, a porosity analysis, an XRD analysis and a mechanical test on compression were made. 

For this, Figure 9 shows the morphology of the sample with the most compact and homogeneous aspect, the least rough surface, and with visible connecting “bridges” on surface fine microrelief. 

Compared to Figure 4, Figure 5, Figure 6 and Figure 7, the samples in Figure 8 and Figure 9 have more SEM images presented, with different magnifications, due to the SEM measurements of the average porosity size performed. As a result, it can be estimated that the average porosity dimension for the sample from Figure 8, with E = 139 J/mm^3^, is slightly higher than for the sample from Figure 9, with E = 100 J/mm^3^. The required final value of an imposed porosity has not yet been established at this stage of the experimental work. This aspect will be considered in future experimental trials. The only correlation that can be made at this stage is between the relative level of porosity and the mechanical toughness that preserves the robustness of the sample. 

However, a porosity analysis was made on the sample from Figure 9 using the method with xylene. The initial mass of the analyzed sample was m_0_ = 0.4387 g. The density of the xylene was 0.866 g/cm^3^. After immersing the sample in xylene, the results of the determinations of the mass of the sample saturated in xylene were as follows: the mass of the sample weighed in air was m_1_ = 0.6292 g, and the one weighed in xylene was m_2_ = 0.2227 g. Based on these determinations, the apparent porosity (P_ap_) of 0.93% and an open porosity (P_o_) of 46.86% were calculated. In-depth porosity analyses are considered for future research experiments, when they can be correlated with an imposed standard level of these porosity values that can assure a concordance with the porous structure of the cortical bone. 

**Figure 10:**
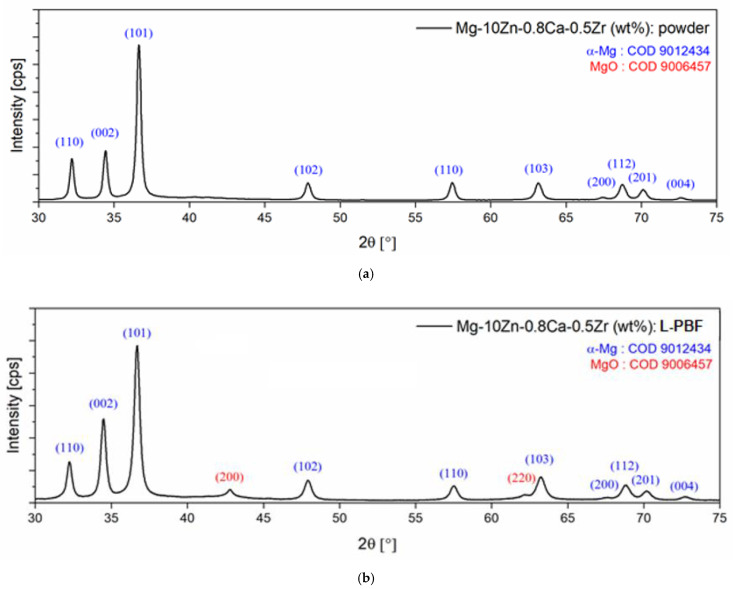
XRD images of the Mg-10Zn-0.8Ca-0.5Zr alloy: (**a**) the initial powder alloy; (**b**) the L-PBF-processed sample.

The microstructural analysis based on SEM images was correlated with the XRD analysis made on the initial powder alloy and on the L-PBF-processed sample with E = 100 J/mm^3^ as well. Figure 10 indicates the XRD patterns corresponding to these two stages of the experiments. The initial powder alloy (Figure 10a) consists of the α-Mg phase only, with no other peaks detected. The α-Mg phase, with diffraction peaks marked (101), (002) and (110) as most intense and with smaller peaks (102), (103), (112), (200), (201) and (004), represents a solid solution indexed in the hexagonal crystalline system, space group 194 (P63/mmc). The corresponding lattice parameters were very close to those of pure Mg, even if there were some minor distortions in crystallographic network. The detection of only the α-Mg phase proves, firstly, that the obtained powder alloy used for L-PBF trials was not contaminated with oxygen during initial mechanical alloying procedure due to the used 1.5 bar high-purity argon protective atmosphere. Secondly, it proves that even if the content of the Zn from the powder alloy (10% wt.) exceeded the solubility limit of Zn in Mg (6.2%wt. at 340 °C and much lower at ambient temperature), the detection of the phase α-Mg only indicated that a super-saturated solid solution was obtained, without traces of a secondary phase such as γ-MgZn_2_. Considering that the powder alloy was obtained through MA, which implies repeated severe plastic deformations of the powder particles with cold welds and constant fractures afterwards, the necessary energy to reach a homogeneous microstructure was obtained, with a similar composition as the proportion of starting constituent powders. This possible result was also reported in [30]. 

Concerning the XRD patterns of the L-PBF sample with E = 100 J/mm^3^ (Figure 10b), it can be observed that, alongside the α-Mg phase, there were also two very small peaks, (200) and (220), corresponding to the chemical compound MgO, having a cubic crystalline system with a lattice parameter of a = 0.4218 nm and space group 225 (Fm-3 m). However, these two diffraction peaks had much lower intensity (were very small and flattened) in comparison with those of the α-Mg phase, indicating that the amount of magnesium oxide formed during L-PBF processing was not significant. The SEM images themselves do not reveal the presence of the MgO, certifying that the amount during this phase was very small. Indeed, the calculated amount was 8.1 wt.% of the MgO, which means that, probably, during the powder manipulation between the end of the MA and start of the L-PBF some oxygen contamination was possible. 

**Figure 11:**
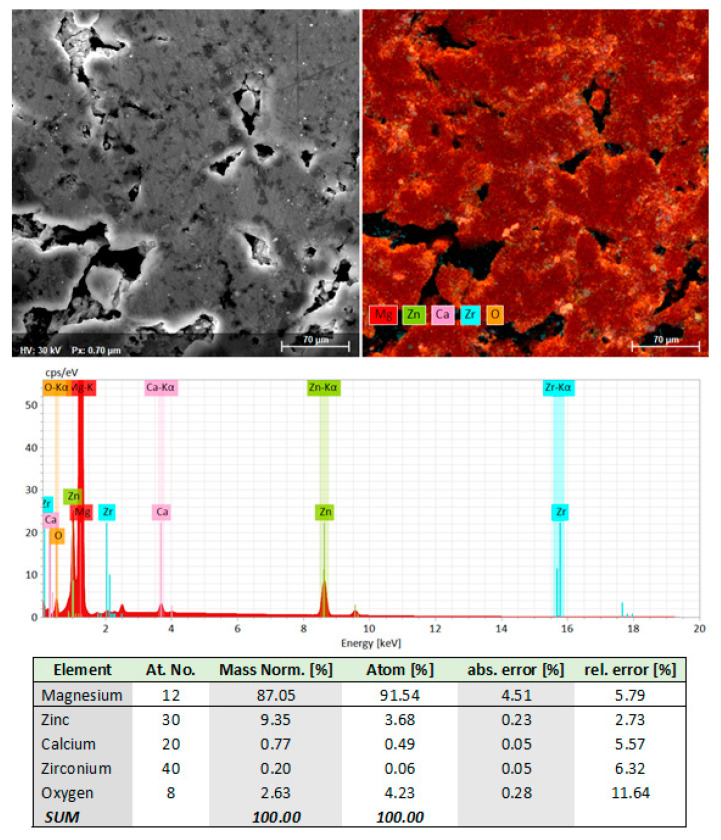
The SEM-EDS image and compositional results for the L-PBF-processed Mg-10Zn-0.8Ca-0.5Zr alloy.

The microstructural analysis of the L-PBF sample was completed with an SEM-EDS analysis to certify the detected phases on the XRD images and the obtained chemical composition. The SEM-EDS examination was made on a polished surface obtained by conventional metallographic procedures. Figure 11 shows that the initial chemical composition of the MA powder alloy was retained after L-PBF processing, with very little oxygen contamination, probably caused during surface polishing for the metallographic analysis. It can also be seen that the distribution of the alloying elements was uniform throughout the mass of the sample, which is consistent with the XRD analysis presented above.

**Figure 12:**
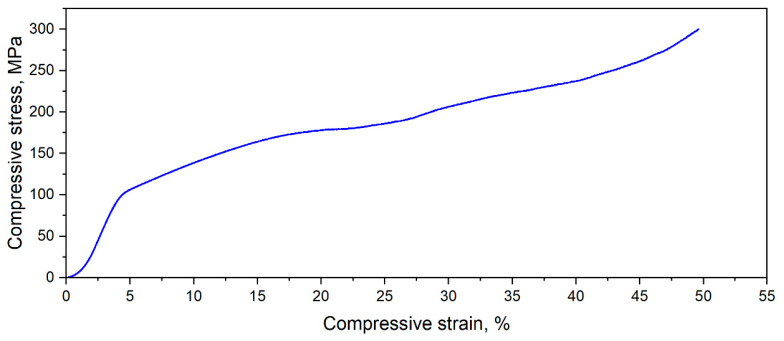
Stress–strain curve corresponding to compressive test applied on L-PBF-processed Mg-10Zn-0.8Ca-0.5Zr alloy with E = 100 J/mm^3^.

In addition to microstructural investigations, the L-PBF-processed Mg-Zn-Ca-Zr alloy samples with the most robust morphology and minor pores were tested for compression. Thus, Figure 12 shows a typical stress–strain curve for a sample in this category. The mechanical testing was performed considering the repeatability and reproducibility of the mechanical characteristics on a batch of five samples. As can be seen in the figure, the compressive yield stress was about 100 MPa and the ultimate compressive strength was 300 MPa, values that are comparable with the 130–200 MPa of the cortical human bone [31,32]. 

## 4. Conclusions

An Mg-10Zn-0.8Ca-0.5Zr alloy was prepared in a solid state using the mechanical alloying of an Mg-Zn-Ca-Zr powder mixture with variation in milling times/milling speed and was then used as a feedstock for L-PBF processing (additive manufacturing).

L-PBF processing parameters varied in large intervals; different parameter combinations (various laser scanning strategies) for 15 distinct variant tests were conducted. The resulting samples were analyzed from a structural point of view (microstructural analysis based on SEM, XRD and porosity analysis) and mechanically tested.

E—the laser energy density [J/mm^3^], an important processing parameter, was used to evaluate the results of the scanning strategies.

The 15 experiments for the additive manufacturing of Mg-10Zn-0.8Ca-0.5Zr (%wt.) revealed that the best sample consistency, with no cracks, good mechanical characteristics (compressive strain) and porosity (the apparent porosity (P_ap_) of 0.93% and an open porosity (P_o_) of 46.86%) was obtained at the lowest applied energy density (E) of 100 J/mm^3^, and also kept in view the correlation of the parameters which contribute to the calculation of the formula.

The E of 100 J/mm^3^ resulted in 45 W laser power (from the tested interval of 45–200 W) and a 600 mm/s scanning speed (from the tested interval 300–1400 mm/s), and these two parameters were considered to have high influence.

The conclusion of this research is that it is necessary to use a low laser power associated with a moderate scanning speed.

This combination of parameters allows for layer adherence with no smog or balling, phenomena which occur at a high laser power.

## Figures and Tables

**Table 1:** The applied L-PBF processing parameters.

Laser Power [W]	Scanning Speed [mm/s]	Layer Height [µm]	Laser Energy Density [J/mm^3^]
45–200	300–1400	20–50	100–579

## Data Availability

The data presented in this study are available on request from the corresponding author.
